# Evaluation of Coagulation Factors and Platelet Activation in Patients Undergoing Complex Endovascular Para-Renal and Thoraco-Abdominal Aneurysm Repair: The Protocol of a Prospective Observational Study

**DOI:** 10.3390/jcm14093105

**Published:** 2025-04-30

**Authors:** Maria P. Ntalouka, Konstantinos Spanos, Paraskevi Kotsi, Aikaterini Bouzia, Georgios Kouvelos, Diamanto Aretha, Efthymia Petinaki, Athanasios Giannnoukas, Miltiadis Matsagkas, Eleni M. Arnaoutoglou

**Affiliations:** 1 Department of Anaesthesiology, Faculty of Medicine, School of Health Sciences, University of Thessaly, Larissa University Hospital, 41110 Larissa, Greece; maria.ntalouka@icloud.com (M.P.N.);; 2 Department of Vascular Surgery, Faculty of Medicine, School of Health Sciences, University of Thessaly, Larissa University Hospital, 41110 Larissa, Greece; 3 Department of Transfusion Medicine, Faculty of Medicine, School of Health Sciences, University of Thessaly, Larissa University Hospital, 41110 Larissa, Greece; 4 Department of Anaesthesiology & Intensive Care, University Hospital of Patras, Rion, 26504 Patras, Greece; 5 Department of Microbiology, Faculty of Medicine, School of Health Sciences, University of Thessaly, Larissa University Hospital, 41110 Larissa, Greece

**Keywords:** aortic aneurysm, thoraco-abdominal, endovascular aneurysm repair, perioperative care, platelet activation, acute kidney injury

## Abstract

**Background/Objectives:** Endovascular aneurysm repair (EVAR) of the aorta may trigger an inflammatory response that affects coagulation. In the EVAR of para-renal and thoraco-abdominal aortic aneurysms, the implants are more complex and the duration of surgery is longer. However, the exact pathophysiological mechanisms of coagulation activation are not yet well understood. The primary aim of this study is to investigate the effects of complex EVAR of para-renal and thoraco-abdominal aortic aneurysms on the coagulation status of patients. **Methods:** This prospective observational study (STROBE), approved and registered by the Ethics Committee of the University Hospital of Larissa (UHL) (NCT06432387), will enroll consecutive patients undergoing elective EVAR of para-renal and thoraco-abdominal aortic aneurysms. Exclusion criteria: Refusal to participate, previous surgery within 3 months, American Society of Anesthesiologists physical status (ASA PS) > 3, known history of thrombophilia or functional platelet dysfunction. Perioperative laboratory tests will be performed according to institutional guidelines. These include a complete blood count, conventional coagulation tests, and kidney and liver function tests. In addition, the following parameters will be determined: von Willebrand factor, factors VIII and XI, D-dimers, fibrinogen, Adamts-13, anti-Xa, platelet activation (multiplate), and high-sensitivity troponin. Blood samples will be taken pre-operatively before induction of anesthesia (01), on postoperative day 1 (02), and on postoperative day 3–4 (03). During hospitalization, myocardial injury after non-cardiac surgery (MINS), major adverse cardiovascular events after non-cardiac surgery (MACE), acute kidney injury (AKI), post-implantation syndrome (PIS), and death from any cause will be recorded. In addition, our patients will be reviewed at 30 days, 3, 6, and 12 months for MACE, implant failure, or death from any cause. All enrolled patients will be treated by the same medical team at UHL according to the indications. According to our power analysis, for a cohort of patients with three consecutive measurements, 58 patients should be included in the study. To compensate for possible dropouts, the sample size was increased to 65 patients. **Conclusions:** The results of the present study could help physicians to better understand the effects of complex EVAR of para-renal and thoraco-abdominal aortic aneurysms on blood coagulation and platelet activation.

## 1. Introduction

### 1.1. Background and Rationale

The development of endovascular techniques in recent decades has changed the treatment of aortic aneurysms [1]. For patients with complex aortic disease, including pararenal abdominal aortic aneurysms (PRAAA) and thoraco-abdominal aortic aneurysms (TAAA), complex endovascular aneurysm repair (EVAR) with the fenestrated or branched EVAR technique (F/BEVAR) was introduced as early as 2012 [1,2,3]. Complex EVAR enables the safe minimally invasive treatment of complex aortic disease in patients with appropriate anatomy and perioperative risk [2,3].

Compared to traditional open surgery (OS), EVAR is characterized by a more favorable short-term outcome in terms of mortality and morbidity in high-risk patients [1,4]. However, despite this, EVAR and thoracic EVAR (TEVAR) appear to be associated with a prominent prothrombotic coagulopathy according to several reports [4,5,6,7,8]. It should be highlighted that the pro-coagulant activity of EVAR and TEVAR is greater when compared with open procedures, and it may lead to higher morbidity due to micro- and macro-vascular thrombosis [5,6,9].

Over the years, EVAR has been associated with increased platelet consumption, increased thrombin activity, increased thrombin generation, and increased fibrinolysis [10]. On the other hand, TEVAR has been associated with increased postoperative platelet consumption and elongation of prothrombin time [6]. Moreover, in contrast to open aneurysm repair (where the aortic thrombus is removed), it has been suggested that the resultant coagulopathy may persist for several months after endovascular procedures, and this may contribute to the development of an aortic thrombus [11].

As far as platelet alterations are concerned, the presence of an abdominal aortic aneurysm (AAA) along the endovascular procedure exhibits a strong impact on platelets [8]. AAA leads to a chronic pro-inflammatory response, which is characterized by increased platelet activation and decreased platelet count [8]. The post-inflammatory response following EVAR exaggerates the decrease in platelet count; however, there are still controversies regarding its impact on platelet activity [8]. Of note, there is a paucity of data regarding the impact of complex EVAR on platelets.

Intraoperative injury to the vascular endothelium, the use of endovascular tools, the mechanical manipulation of the intramural thrombus of the aneurysm during stent–graft implantation, the manipulation of guidewires and catheters in the vessels, the activation of platelets by the graft material, and the use of contrast media have all been recognized as the possible contributing factors of coagulation changes in patients undergoing EVAR procedures [4,5,6,7,8,9,10,12]. The manipulation of guidewires and catheters in the vessels may enhance the intraoperative injury of the vascular endothelium, leading to increased thrombin generation and activity [10,13,14]. Furthermore, contrast media can be linked with platelet activation and endothelial cell injury [10,15,16]. Moving on to intramural sac thrombus, in contrast to OS, the intramural sac thrombus is not removed during an EVAR procedure. It has been suggested that the remanence of an intramural thrombus after EVAR could serve as a possible mechanism for persistent (up to 12 months) hypercoagulability, when compared to OS [10,13,17]. Normally, the exclusion of the aneurysm sac from the circulation leads to complete sac thrombosis with subsequent platelet consumption, increased coagulation, and fibrinolysis activation [10]. In type II endoleak, only partial sac thrombosis occurs; the aneurysm thrombus remains in situ and in contact with the systemic circulation. However, notably, even in the absence of a clinically significant endoleak, the thrombus could still remain in contact with the systemic circulation through the small feeding lumbar arteries or the mesenteric circulation [10,13,17].

### 1.2. Aims

To our knowledge, no specific analysis of coagulation changes or platelet activation after complex EVAR has been performed to date. Considering the high complexity of the repair and the longer stent graft in complex EVAR, we decided to assess the hypothesis that the complex EVAR of para-renal and thoraco-abdominal aortic aneurysms might induce a more severe coagulation response along with a more prominent platelet activation.

### 1.3. Objectives

(1) To evaluate the early (postoperative day one) and late (postoperative day 3 or 4) effects of complex EVAR of para-renal and thoraco-abdominal aortic aneurysms on coagulation, with respect to von Willebrand factor (vWF), factors VIII and XI, D-dimers, fibrinogen, Adamts-13 levels, and platelet activation, in patients undergoing complex para-renal and thoraco-abdominal aortic aneurysm repair (primary endpoint);
(2) To record the incidence of myocardial injury after non-cardiac surgery (MINS), major adverse cardiovascular events after non-cardiac surgery (MACE), acute kidney injury (AKI), post-implantation syndrome (PIS), and death from any cause during hospitalization;
(3) To record the incidence of MACE, implant failure, or death from any cause at 30 days and 3, 6, and 12 months postoperatively.

## 2. Materials and Methods

### 2.1. Experimental Design

This is a prospective observational study that will be conducted at a single tertiary/referral University Hospital using the STROBE guidelines (Strengthening the Reporting of Observational Studies in Epidemiology) [18].

### 2.2. Study Setting

**Figure 1:**
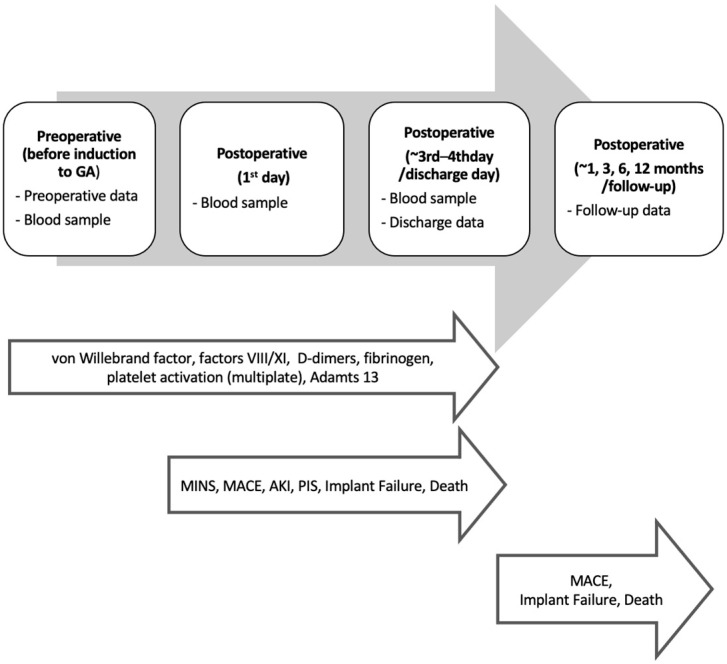
Study timeline.

This prospective observational study will be conducted in a tertiary institution, i.e., the Larissa University Hospital (UHL). The study plan and data collection are summarized in Figure 1.

### 2.3. Study Schedule

Preoperatively and before induction to general anesthesia, the preoperative data, including demographics (age, gender, body mass index), American Society of Anesthesiologists physical status (ASA PS), any comorbidities and administrated drug therapy, smoking status and alcohol intake, the size of aneurysm, the type of graft used, and baseline liver and renal function will be recorded. In addition, a complete blood count, conventional coagulation tests, kidney and liver function tests, vWF, factors VIII and XI, D-dimers, fibrinogen, Adamts-13, platelet activation (multiplate), and high-sensitivity troponin will be obtained. The anti-Xa value will also be determined in order to rule out an anticoagulant effect on the laboratory tests.

At the first postoperative day, the duration of surgery and the possible occurrence of MINS, MACE, AKI, and PIS will be recorded, while a complete blood count, conventional coagulation tests, kidney and liver function tests, vWF, factors VIII and XI, D-dimers, fibrinogen, Adamts-13, platelet activation (multiplate), and high-sensitivity troponin will be obtained. The anti-Xa value will also be determined in order to rule out an anticoagulant effect on the laboratory tests.

At the 3rd or 4th postoperative day, based on the date of discharge, the length of hospitalization, the number of transfusion units, and the possible occurrence of MINS, MACE, AKI, and PIS will be recorded. In addition, a complete blood count, conventional coagulation tests, kidney and liver function tests, vWF, factors VIII and XI, D-dimers, fibrinogen, Adamts-13, platelet activation (multiplate), and high-sensitivity troponin will be obtained from a peripheral vein. The anti-Xa value, correlated to the weight and the dose of the anticoagulant, is also determined in order to rule out an anticoagulant effect on the laboratory tests.

At 1, 3, 6, and 12 months postoperatively, MACE, implant failure (including secondary sac rupture, graft explantation, type 1 or 3 endoleak, or major mitigation of the stent grafts), and death from any cause will be recorded. The study plan and data collection are summarized in Figure 1.

### 2.4. Participants

Consecutive patients with para-renal and thoraco-abdominal aortic aneurysms undergoing complex EVAR will be included. All patients will be followed up for 12 months. In general, patients will be treated according to the guidelines of the European Society for Vascular Surgery (ESVS) [19].

### 2.5. Eligibility Criteria

Consecutive adult patients, 18 years or older, with para-renal and thoraco-abdominal aortic aneurysms undergoing complex EVAR will be included.

Patients that refuse to participate, those who have undergone any prior surgery within 3 months, those with history of inherited or acquired bleeding disorder, those with inherited or acquired platelet dysfunction, those with factor V Leiden or any type of thrombophilia, antiphospholipid syndrome, protein C or S deficiency, or patients with an active malignancy or a connective tissue or rheumatoid disease and patients classified as ASA PS > 3 will be excluded. Inclusion and exclusion criteria are presented in Table 1 and Table 2.

### 2.6. Standard Perioperative Care

Perioperative care will be provided in accordance with institutional guidelines and the updated Clinical Practice Guidelines on the Management of Abdominal Aorto-iliac Artery Aneurysms [19]. All patients will be treated by the same surgical and anesthesiologic team in a fully equipped operating theatre under general anesthesia. Intravenous induction to general anesthesia will be achieved with propofol and maintenance with desflurane and dexmedetomidine. In addition to standard monitoring (electrocardiogram, blood pressure, and pulse oximeter), depth of anesthesia, invasive blood pressure, and goal-directed fluid-therapy monitoring will be applied. Every effort will be made to follow the selection criteria recommended by the stent graft manufacturer. However, the final decision will be made by the treating surgeon.

Preoperatively, patients will be treated with 100 mg of aspirin once daily for at least 5 days. If the patient has been treated with clopidogrel, this will be switched to aspirin preoperatively for 10 days [20]. Postoperatively, patients will receive 100 mg of aspirin if there is no bleeding and 75 mg of clopidogrel if there is no neurological dysfunction due to spinal ischemia and dual antiplatelet therapy (DAPT, aspirin 100 mg and clopidogrel 75 mg) from the next day. If the patient has been treated with anticoagulants, these will be discontinued preoperatively in accordance with the ACCP guidelines and resumed on postoperative day 1 or 2, depending on hemostasis [20]. In this case, patients will be treated preoperatively with aspirin 100 mg once daily for 5 days, and aspirin will be continued postoperatively.

Systemic heparinization of the patient will be achieved with 5000 units of unfractionated heparin intraoperatively. One hour after the first administration of heparin, and every 30 min thereafter, the activated clotting time (ACT) will be measured. The intraoperative ACT target is set at 200–300 s.

### 2.7. Outcomes

The primary outcome is to evaluate the effects of complex EVAR of para-renal and TAAA on coagulation with respect to von Willebrand factor (vWF), factors VIII and XI, D-dimers, fibrinogen, and Adamts-13 levels and on platelet activation (multiplate).

Perioperative laboratory tests will be performed according to institutional guidelines. These include a complete blood count, conventional coagulation tests, and kidney and liver function tests. In addition, the following parameters will be determined: vWF, factors VIII and XI, D-dimers, fibrinogen, Adamts-13, platelet activation (multiplate), and high-sensitivity troponin (hs-cTNT). The blood samples will be taken at three points in time: preoperatively before induction to general anesthesia, early postoperatively (1st postoperative day), and late postoperatively (3rd–4th postoperative day). The anti-Xa value, correlated to the weight and the dose of the anticoagulant, will also be determined in order to rule out an anticoagulant effect on the laboratory tests.

MINS, MACE, AKI, PIS, implant failure, and death from any cause during hospitalization were defined as secondary outcomes. MACE, implant failure (including secondary sac rupture, graft explantation, type 1 or 3 endoleak or major mitigation of the stent grafts), and death from any cause at 1, 3, 6, and 12 months postoperatively were also defined as secondary endpoints [21].

MINS is defined as postoperative hs-cTNT concentration 20 to 65 ng/L with an absolute change of >/=5 ng/L or any postoperative absolute value >/=65 ng/L [22,23].

MACE is defined as myocardial infarction (any clinical symptom associated with an acute coronary syndrome and/or any new electrocardiographic sign or high-sensitivity troponin elevation), arrhythmia (any event of atrial or ventricular tachycardia with more than 90 pulses per minute or any episode of bradycardia of less than 50 pulses per minute), and stroke (any transient ischaemic attack, any stroke: major or minor according to Rankin Score) [24].

PIS is defined as the presence of at least two of the systemic inflammatory response syndrome (SIRS) criteria, including fever >38 °C and leucocytosis >12,000/μL and obvious clinical or biochemical sign of infection (negative urine and blood cultures and chest X-ray) [24].

AKI is defined according to the RIFLE criteria (Risk, Injury, Failure, Loss of kidney function, and End-stage kidney disease), as a twofold increase in serum creatine or >50% decrease in GFR (estimated using the Cockcroft–Gault equation) [24].

### 2.8. Data Collection

Most of the data will be obtained from the routine admission documents and the electronic patient file. They will be recorded in a password-protected spreadsheet. Basic data collection will include demographics (age, gender, body mass index), ASA physical status, any comorbidities and administrated drug therapy, smoking status and alcohol intake, the size of aneurysm, the type of graft, liver and renal function, the duration of surgery, the length of hospitalization, the number of transfusion units, and the possible occurrence of MINS, MACE, AKI, and PIS. The basic data that will be collected are presented in Table 3.

As far as the laboratory values are concerned, the following parameters will be determined: vWF, factors VIII and XI, D-dimers, fibrinogen, Adamts-13, platelet activation (multiplate), and high-sensitivity troponin. All samples will be taken by puncturing a peripheral vein. The blood samples will be taken at three points in time: preoperatively before induction of general anesthesia, early postoperatively (1st postoperative day), and late postoperatively (3rd–4th postoperative day). The anti-Xa value, correlated to the weight and the dose of the anticoagulant, will also be determined in order to rule out an anticoagulant effect on the laboratory tests. The additional laboratory values that will be collected are presented in Table 4.

## 3. Statistical Methods

### 3.1. Sample Size

Our study will include one group arm. According to our power analysis (F-test, Anova Repeated Measures, within factors), for a cohort of patients with three consecutive laboratory measurements, considering an effect size = 0.25, a = 0.05, and 95% power, 58 patients should be included in the study. To compensate for possible dropouts, the sample size was increased to 65 patients.

### 3.2. Statistical Analysis

The normality of our data will be tested with both the Shapiro–Wilk and the Kolmogorov–Smirnov tests. Fisher’s exact and chi-square tests will be used for the evaluation of differences between categorical variables, and the data will be presented as frequencies and percentages. Continuous variables, following a normal distribution, will be tested with the student’s *t*-test and will be presented as mean and standard deviation (SD), while the Mann–Whitney U test will be used for those without a normal distribution, and data will be presented as median and interquartile range (IQR). Anova Repeated Measures, parametric or nonparametric test, as appropriate, will be used for the evaluation of possible differences among parameters measured at different time points.

A univariate and multivariate analysis will be conducted using linear or logistic regression, as appropriate, with backward stepwise elimination for the assessment of independent risk factors contributing to our primary outcome. Our final multivariable model will include variables with *p*-values < 0.1 in the univariate regression, while the choice of variables will also be based on scientific knowledge. Variance inflation factor (VIF) will be used for the assessment of multicollinearity issues. Independent factors contributing to our primary outcome will be presented as odds ratio (O.R.) or regression coefficient and 95% confidence interval (CI) as appropriate.

Sensitivity and specificity analyses will also be performed using the Receiver Operating Characteristic Curves (ROC). Data will be analyzed using the Statistical Package for the Social Sciences (SPSS, version 28.0; IBM, Armonk, NY, USA) and GraphPad Prism Software (La Jolla, CA, USA, version 10.4.4), and *p*-values < 0.05 will be considered significant.

## 4. Ethics and Dissemination

The study has been approved by the Ethical Committee of Larissa University Hospital (22438-27 May 2024). All patients will be informed verbally and in writing. In accordance with the Declaration of Helsinki, written informed consent will be obtained from all participating patients. For patients who are unable to provide informed consent, their next of kin will provide informed consent [25]. Participants’ data will be pseudo-anonymized with a study identification (ID) [18]. This study has been registered at clinicaltrials.org (NCT06432387, registered in May 2024).

## 5. Expected Results

By conducting this prospective study, we aim to investigate the effects of complex EVAR of para-renal and TAAA on blood coagulation with regard to vWF, factors VIII and XI, D-dimers, fibrinogen, and Adamts-13 and platelet activation (multiplate) in patients undergoing complex para-renal and thoraco-abdominal aortic aneurysm repair. In addition, the incidence of MINS, MACE, AKI, PIS, implant failure, and death from any cause will be investigated in patients undergoing complex EVAR of para-renal and TAAA. Moreover, we believe that the results of our study could guide the design of future studies in this high-risk population, where high-quality data are still lacking.

## 6. Strengths and Limitations

The fact that the study will be conducted at a single center, with one group arm, and the fact that certain laboratory values will be assessed should be considered as the main limitations. However, the study design was based on any available means and markers from our hospital. Moreover, to the best of our knowledge, this is the first study to investigate the aforementioned specific coagulation factors, including Adamts-13, in the perioperative setting in complex endovascular aneurysm repair of para-renal and thoraco-abdominal aortic aneurysms.

## 7. Conclusions

In summary, the results of the present study could help physicians to better understand the impact of complex para-renal and thoraco-abdominal EVAR on blood coagulation and platelet activation.

## 8. Patients’ Involvement and Dissemination Strategy

The study findings will be submitted for publication in peer-reviewed journals and will be presented at scientific meetings.

## Figures and Tables

**Table 1:** Inclusion Criteria.

Inclusion Criteria
Age > 18 years
Consecutive patients undergoing complex thoraco-abdominal aortic aneurysm repair in UHL

**Table 2:** Exclusion Criteria.

Exclusion Criteria
Refuse to participate
Prior surgery within 3 months
History of inherited or acquired bleeding disorder
History of inherited or acquired platelet dysfunction
Patients with factor V Leiden or any type of thrombophilia
Patients with antiphospholipid syndrome
Patients with protein C or S deficiency
Patients with connective tissue or rheumatoid disease
Patients with an active malignancy
ASA PS > 3

**Table 3:** Basic Data Collection.

Basic Data Collection	
Demographics: age, gender, body mass index	MINS
ASA PS	MACE
Comorbidities and administrated drug therapy	AKI
Lifestyle: smoking status, alcohol intake	PIS
Size of aneurysm	Duration of surgery
Type of graft	Length of hospitalization
Liver and renal function	Transfusion units

MINS: myocardial injury after non-cardiac surgery, MACE: major adverse cardiovascular events after non-cardiac surgery, AKI: acute kidney injury, PIS: post-implantation syndrome (PIS).

**Table 4:** Laboratory values.

Laboratory Values	
vWF	Factor VIII
Factor XI	D-dimers
Fibrinogen	Adamts-13
Platelet function (multiplate R)	Anti-Xa

## Data Availability

The datasets of the current study can be requested from the corresponding author after completion of the study upon reasonable request.

## References

[1] Conroy P.D., Rastogi V., Yadavalli S.D., Solomon Y., Romijn A.S., Dansey K., Verhagen H.J.M., Giles K.A., Lombardi J.V., Schermerhorn M.L. (2025). The rise of endovascular repair for abdominal, thoracoabdominal, and thoracic aortic aneurysms. J. Vasc. Surg..

[2] Xodo A., D’Oria M., Mendes B., Bertoglio L., Mani K., Gargiulo M., Budtz-Lilly J., Antonello M., Veraldi G.F., Pilon F. (2022). Peri-Operative Management of Patients Undergoing Fenestrated-Branched Endovascular Repair for Juxtarenal, Pararenal and Thoracoabdominal Aortic Aneurysms: Preventing, Recognizing and Treating Complications to Improve Clinical Outcomes. J. Pers. Med..

[3] Oderich G.S., Forbes T.L., Chaer R., Davies M.G., Lindsay T.F., Mastracci T., Singh M.J., Timaran C., Woo E.Y., Writing Committee Group (2021). Reporting standards for endovascular aortic repair of aneurysms involving the renal-mesenteric arteries. J. Vasc. Surg..

[4] Chang C.K., Chuter T.A., Niemann C.U., Shlipak M.G., Cohen M.J., Reilly L.M., Hiramoto J.S. (2009). Systemic inflammation, coagulopathy, and acute renal insufficiency following endovascular thoracoabdominal aortic aneurysm repair. J. Vasc. Surg..

[5] Arnaoutoglou E., Kouvelos G., Papa N., Karamoutsios A., Bouris V., Vartholomatos G., Matsagkas M. (2016). Platelet activation after endovascular repair of abdominal aortic aneurysm. Vascular.

[6] Kahlberg A., Rinaldi E., Tshomba Y., Spelta S., Mascia D., Melissano G., Chiesa R. (2018). Volumetric analysis of aneurysm thrombosis after thoracic endovascular aortic repair predicts postoperative changes in platelet count and coagulation parameters. J. Cardiovasc. Surg..

[7] Sun W., Zheng J., Gao Y. (2022). Targeting Platelet Activation in Abdominal Aortic Aneurysm: Current Knowledge and Perspectives. Biomolecules.

[8] Burban A., Idzik A., Gelo A., Filipiak K.J., Jakimowicz T., Jama K., Grabowski M., Gasecka A., Siniarski A. (2022). Platelet function changes in patients undergoing endovascular aortic aneurysm repair: Review of the literature. Front. Cardiovasc. Med..

[9] Englberger L., Savolainen H., Jandus P., Widmer M., Do do D., Haeberli A., Baumgartner I., Carrel T.P., Schmidli J. (2006). Activated coagulation during open and endovascular abdominal aortic aneurysm repair. J. Vasc. Surg..

[10] Kapetanios D.M., Karkos C.D., Papazoglou K.O. (2018). Changes in circulating markers of coagulation and fibrinolysis after EVAR. Int. Angiol..

[11] Aho P.S., Niemi T., Piilonen A., Lassila R., Renkonen R., Lepäntalo M. (2007). Interplay between coagulation and inflammation in open and endovascular abdominal aortic aneurysm repair--impact of intra-aneurysmal thrombus. Scand. J. Surg..

[12] Michalska M., Grochowiecki T., Wyczałkowska-Tomasik A., Pączek L., Jakimowicz T., Cacko A., Jama K., Stec A., Sikorska E., Nazarewski S. (2023). Evaluation of selected parameters of inflammation, coagulation system, and formation of extracellular neutrophil traps (NETs) in the perioperative period in patients undergoing endovascular treatment of thoracoabdominal aneurysm with a branched device (t-Branch). Front. Cardiovasc. Med..

[13] Namiki A., Toma H., Nakamura M., Matsuda K., Hara H., Hara H., Asahara T., Soumitsu Y., Kobayashi N., Yamaguchi T. (2004). Hemostatic and fibrinolytic activation is less following cutting balloon angioplasty of the coronary arteries. Jpn. Heart. J..

[14] Tschopl M., Tsakiris D.A., Marbet G.A., Labs K.H., Jäger K. (1997). Role of hemostatic risk factors for restenosis in peripheral arterial occlusive disease after transluminal angioplasty. Arterioscler. Thromb. Vasc. Biol..

[15] Serino F., Abeni D., Galvagni E., Sardella S.G., Scuro A., Ferrari M., Ciarafoni I., Silvestri L., Fusco A. (2002). Noninvasive diagnosis of incomplete endovascular aneurysm repair: D-dimer assay to detect type I endoleaks and nonshrinking aneurysms. J. Endovasc. Ther..

[16] Kapetanios D., Karkos C.D., Pliatsios I., Mitka M., Giagtzidis I.T., Konstantinidis K., Papazoglou K.O. (2019). Association Between Perioperative Fibrinogen Levels and the Midterm Outcome in Patients Undergoing Elective Endovascular Repair of Abdominal Aortic Aneurysms. Ann. Vasc. Surg..

[17] Bailey M.A., Griffin K.J., Sohrabi S., Whalley D.J., Johnson A.B., Baxter P.D., Ariëns R.A., Scott D.J. (2013). Plasma thrombin-antithrombin complex, prothrombin fragments 1 and 2, and D-dimer levels are elevated after endovascular but not open repair of infrarenal abdominal aortic aneurysm. J. Vasc. Surg..

[18] von Elm E., Altman D.G., Egger M., Pocock S.J., Gøtzsche P.C., Vandenbroucke J.P., STROBE Initiative (2008). The Strengthening the Reporting of Observational Studies in Epidemiology (STROBE) statement: Guidelines for reporting observational studies. J. Clin. Epidemiol..

[19] Wanhainen A., Verzini F., Van Herzeele I., Allaire E., Bown M., Cohnert T., Dick F., van Herwaarden J., Karkos C., Koelemay M. (2019). Editor’s Choice—European Society for Vascular Surgery (ESVS) 2019 Clinical Practice Guidelines on the Management of Abdominal Aorto-iliac Artery Aneurysms. Eur. J. Vasc. Endovasc. Surg..

[20] Douketis J.D., Spyropoulos A.C., Murad M.H., Arcelus J.I., Dager W.E., Dunn A.S., Fargo R.A., Levy J.H., Samama C.M., Shah S.H. (2022). Perioperative Management of Antithrombotic Therapy: An American College of Chest Physicians Clinical Practice Guideline. Chest.

[21] Harrison S.C., Winterbottom A.J., Coughlin P.A., Hayes P.D., Boyle J.R. (2018). Editor’s Choice—Mid-term Migration and Device Failure Following Endovascular Aneurysm Sealing with the Nellix Stent Graft System—A Single Centre Experience. Eur. J. Vasc. Endovasc. Surg..

[22] Chew M.S., Saugel B., Lurati-Buse G. (2023). Perioperative troponin surveillance in major noncardiac surgery: A narrative review. Br. J. Anaesth..

[23] Hughes C., Ackland G., Shelley B. (2024). Perioperative myocardial injury. BJA Educ..

[24] Ntalouka M.P., Nana P., Brotis A., Chatzis A., Mermiri M., Stamoulis K., Bareka M., Giannoukas A., Matsagkas M., Arnaoutoglou E. (2023). Predictors of 30-Day Postoperative Outcome after Elective Endovascular Abdominal Aortic Aneurysm Repair: A Tertiary Referral Center Experience. J. Clin. Med..

[25] World Medical Association (2013). World Medical Association Declaration of Helsinki: Ethical principles for medical research involving human subjects. JAMA.

